# Intersectional inequalities in exclusive breastfeeding practices in India: analysis of national family health survey-4

**DOI:** 10.1186/s13006-023-00577-x

**Published:** 2023-08-23

**Authors:** Haseena Chekrain Valappil, Rajeev Jayalakshmi, Christian Sewor

**Affiliations:** 1 Department of Public Health and Community Medicine, Central University of Kerala, Tejaswini Hills, Periye, 671320 Kasaragod, Kerala India; 2 Public Health Research Group, Department of Biomedical Sciences, University of Cape Coast, Cape Coast, Ghana

**Keywords:** Exclusive breastfeeding, Intersectionality, Infants, India, Inequalities

## Abstract

**Background:**

Exclusive breastfeeding in the initial six months of infancy plays a significant role in the physical and cognitive development of the child. One in two children below six months of age in India is not receiving exclusive breastfeeding, with the rates varying considerably between and within states. In this study, we investigated the effect of intersecting inequalities in exclusive breastfeeding practice amongst children below six months in India.

**Methods:**

Data from the fourth National Family Health Survey (NFHS-4) was used for the study. The study used a weighed sample of 211,145 infants below six months. Exclusive breastfeeding practice was assessed based on the previous 24-hours feeding practice of the child. Intersecting social categories were created based on place of residence, religion, wealth index, and mothers’ education. A binary logistic regression model was used to explore inequalities in the practice of exclusive breastfeeding based on the intersecting social categories.

**Results:**

Exclusive breastfeeding practices varied significantly between the intersecting categories of religion, place of residence, wealth index, and education of the mother. Exclusive breastfeeding practice prevalence was the highest amongst children born in the Urban-Secondary-Poor-Others group (57.9%) and lowest amongst the Rural-Primary-Rich-Others category (34.5). In comparison to children in the most disadvantaged category (Rural-Primary-Poor-Others), children born in the Rural-Secondary-Poor-Others category had the highest odds [OR (odds ratio) 1.213; 95% CI 1.024, 1.437] of being exclusively breastfed, whilst children within the Rural-Primary-Rich-Others category had the lowest odds (OR 0.494; 95% CI 0.345, 0.708). Wide disparities were observed in the odds of engaging in exclusive breastfeeding practice amongst the middle groups than between the most advantaged and the most disadvantaged groups. The inequality indices show varied distribution of exclusive breastfeeding prevalence across the intersecting groups with higher exclusive breastfeeding prevalence noted amongst disadvantaged groups.

**Conclusions:**

The study found that intersecting inequalities in exclusive breastfeeding exist in India. In order to improve exclusive breastfeeding practice, targeted interventions must acknowledge and adopt a comprehensive approach that addresses inherent inequalities resulting from the intersection of various axes of social stratification.

## Background

Exclusive breastfeeding (EBF) is a crucial health intervention that helps facilitate the growth and development of newborn children. The World Health Organization (WHO) estimated that nearly 820,000 children could be saved every year with exclusive breastfeeding [1]. Exclusive breastfeeding reduces infant mortality and morbidity by facilitating proper immune development and protecting against common childhood diseases like diarrhoea, gastrointestinal infections, pneumonia, and allergies [1, 2]. Intelligence or cognitive scores of the babies have been reported increase with EBF practise, and exclusively breastfed babies are less prone to childhood overweight and obesity [3]. Exclusive breastfeeding is also beneficial to maternal health, as it has been reported to reduce maternal risk of breast and ovarian carcinoma, type 2 diabetes, heart diseases [4]. It helps prevent uterine contraction by increasing oxytocin levels and reduces bleeding during pregnancy [4]. In low- and middle-income countries exclusively breastfed infants have been reported to be associated with a 13% less risk of mortality, compared to their counterparts [5].

Despite the benefits and multiple interventions that promote EBF, its prevalence is unacceptably low globally and varies significantly within and between countries. As per the WHO report in 2018, the global prevalence of EBF is 40% [6]. In 2019, a UNICEF report indicated that about 44% of children were exclusively breastfed globally, with Asian countries reporting a slightly high prevalence of 57% [7]. A previous multi-country study by Cai and colleagues reported that EBF prevalence in developing countries has increased from 33 to 39% over five years [8]. In South Asia, the study reported moderate changes in EBF prevalence (40% in 1995 to 45% in 2010) [8]. Estimates from a multi-country study by Zong et al. [9] reported the global prevalence of EBF amongst children under six months to be 45.7% (45.2%, 46.2%) between 2010 and 2018 with South-East Asia/Western Pacific region having the highest prevalence 55.2% (54.4%, 56.0%). Although this estimate shows improvements in EBF prevalence, with the region meeting the WHO target of 50% by 2025 [10], country-wise estimates indicates wide variability in the EBF prevalence with a country like India yet to meet the target [43.5% (42.9, 44.1) [9].

In India, despite having a relatively high neonatal mortality rate of 32 deaths per 1000 live births, the prevalence of EBF of children below six months stood at 55% in 2016, with only a 9% increase between 2005 and 2015 [11]. Furthermore, there are still regional disparities in the exclusive breastfeeding practice. Among the states and union territories, the highest prevalence of EBF was reported in Chhattisgarh (77.2%) and the lowest in Meghalaya (35.8%) [12]. Among the South Indian states, Andhra Pradesh reported the highest prevalence of exclusive breastfeeding (71.1%), followed by Karnataka (54.2%), Kerala (53.3%), and Tamil Nadu (48.3%) [12].

While India has clear policies, guidelines, and legislation intended to promote infant and young child feeding practices, including exclusive breastfeeding [13], the slow progress in improving EBF prevalence highlights the presence of specific barriers impeding this progress. For instance, since World Health Assembly implemented the International Code of marketing breast milk substitutes in the year 1981 which restricts the advertisements and promotion of breast milk substitutes and promotes EBF, India has had the full provision of this law since 1983 [14]. Also, baby-friendly hospital initiatives were launched in 1992 to promote and support exclusive breastfeeding practice [15].

Studies have reported the disproportionate prevalence of EBF across the multiple axes of social stratification such as religion, social class, maternal education, place of residence, and employment status of the mother [12]. Within the Indian context, various studies have reported that rural residency, living in poorer households, high maternal education, and being from the Hindu religion has a positive effect on exclusive breastfeeding [11, 12, 16–19].

It is worth noting that while there is a consensus on the inequality observed in EBF practices based on the axes of social stratification, no studies have investigated its pathway and the effect of intersectionality on exclusive breastfeeding practices. In the contexts mentioned above, the present study investigates whether the intersection of social axes (religion, place of residence, social class, and maternal education) and resulting social position influence the EBF of children below six months in India.

Research question: Does the intersection of religion, place of residence, education, and social class (wealth index) inequalities influence the exclusive breastfeeding of children in India?

## Methods

### Study design and population

This study was a secondary analysis of the National Family Health Survey (NFHS-4) conducted in 2015–2016. The NFHS is a nationally representative cross-sectional survey using a multistage cluster random sampling. This survey is conducted amongst households, children (age 0–5), women (age 15–49), and men (age 15–54). The main aim of the survey is to collect information on fertility, contraception, infant mortality, maternal and child health, and nutritional assessment at the national level.

### Sample size and sampling procedure

The NFHS-4 included 640 districts in India as per the 2011 Census. A total of 572,000 households,  were selected through a complete mapping of both urban and rural areas. The urban sample was selected through a two-stage sampling, with Census Enumeration Blocks (CEB) being selected in the first stage after which a random selection of 22 households in each CEB are selected. The rural sample was also selected through a two-stage sampling. The villages were taken as the Primary Sampling Units (PSU) in the initial stage, after which 22 households were randomly selected in each PSU. The NFHS-4 has collected data on children below five years. After applying the sample weight, 21,145 children aged below six months were there in the data [11].

### Data collection

Computer-Assisted Personal Interview was the data collection mode. The NFHS-4 fieldwork for India was conducted from 20 January 2015 to 4 December 2016 by 14 Field Agencies and gathered information from 601,509 households, 699,686 women, and 112,122 men. Four types of tools were used in the survey to collect information. It includes the household questionnaire, women’s questionnaire, men’s questionnaire, and biomarker assessment. Questionnaires are available in 19 languages using Computer Assisted Personal Interviewing (CAPI) [11].

### Study variables

Exclusive breastfeeding among children was the outcome variable. Infants less than six months who received only breastmilk except for medicines were regarded as exclusively breastfed [20, 21]. This indicator was assessed based on the youngest child living with the mother and the feeding practice within 24 h before the day of data collection.

The population was the youngest living children aged 0–5 months, living with their mothers (women aged 15–49 years).

As per the DHS coding manual, prevalence of EBF was measured as the proportion of infants aged 0–5 months who received breastmilk as the only source of nourishment (but also received prescribed oral rehydration solution, medicines and vitamin syrups or drops) in the last 24 h before the survey based on mothers’ recall [12, 22]. This measurement was consistent with the WHO/UNICEF guidelines for assessing IYCF practices [23]. Missing data on breastfeeding is treated as not currently breastfeeding in the numerator and included in the denominator [22]. Missing and “don’t know” data on foods and liquids given is treated as not given in the numerator and included in the denominator [22].

In this study, four predictor variables (religion, household wealth, maternal education, and place of residence) which were associated with exclusive breastfeeding as per the existing literature and were more likely to determine the social position of the child were combined to generate intersecting categories which was the main predictor variable. Three other variables (maternal age, caste, and gender of child) served as confounders. Firstly, the selected variables for creating intersecting categories were converted into dichotomous variables as follows.

Religion: Hindu – Hindu & (Muslim, Christian, Buddhist, Sikh, Jain, and others) – Others.

Wealth index: Poorest, poorer, and middle - poor & richer and richest – rich.

Education of mother: Illiterate and primary education – primary & secondary and higher education – secondary.

Then we combined these variables and the place of residence (rural/urban) to create intersecting social categories. There were 16 intersecting categories, as depicted in Table 1.

**Table 1:** Intersecting categories

Urban-Primary-Poor-Hindu	Rural-primary-Poor-Hindu
Urban-Primary-Poor-Others	Rural-Primary-Poor-Others
Urban-Primary-Rich-Hindu	Rural-Primary-Rich-Hindu
Urban-Primary-Rich-Others	Rural-Primary-Rich-Others
Urban-Secondary-Poor-Hindu	Rural-Secondary-Poor-Hindu
Urban-Secondary-Poor-Others	Rural-Secondary-Poor-Others
Urban-Secondary-Rich-Hindu	Rural-Secondary-Rich-Hindu
Urban-Secondary-Rich-Others	Rural-Secondary-Rich-Others

### Data analysis

The data were analyzed using the SPSS Version 27 (univariate and multivariate analysis) and Stata Version 17 MP-Parallel Edition (slope inequality index and graphs). Prior to carrying out the univariate and multivariate analyses, the appropriate sample weights were applied to the data. Descriptive statistics (frequency and percentage) were used to explore the distribution of the sociodemographic factors and EBF prevalence in the population. Binary logistic regression was used to assess the relationship between the intersecting categories and EBF prevalence. The model was subsequently adjusted for maternal age, caste, and sex of the child. Odds ratio with a *p* - value < 0.05 at a 95% confidence interval was considered statistically significant. Model fitness was assessed using the Pearson goodness of fit test.

Inequalities in EBF prevalence across the intersecting groups was assessed using absolute and relative indices. The absolute measure of inequality was derived by computing the difference in EBF prevalence within the most advantaged group and disadvantaged group. The relative disparity in exclusive breastfeeding prevalence was also computed by dividing EBF prevalence among the most advantaged group and disadvantaged group. Slope and relative inequality indices were also obtained via a logistic regression model using the *siilogit* command [24].

### Data access permission and ethical considerations

The National Family Health Survey-4 (Demographic and Health Survey) datasets were available in the public domain, after removing the identifying information. To conduct the study, the first author obtained the Ethics clearance exemption from the Institutional Human Ethics Committee (IHEC), Central University of Kerala (Approval number: IHEC/CUK/2021/15).

## Results

This study included 21,145 children below six months of age from all over India. Of them, about 52% were boys and 48% were girls. Table 2 describes the distribution of variables selected to create intersecting categories. Nearly three fourth of the children belonged to rural areas. Three out of five mothers had secondary level education and above. The majority of children were from the Hindu religion (78.6%). More than two-thirds of the children were born to poor families as per the wealth index measured in NFHS-4.

**Table 2:** Distribution of selected sociodemographic factors

Variable	Frequencies (*n*)	Percentage (%)
**Place of residence (N = 21,145)**		
Urban	5550	26.2
Rural	15,595	73.8
**Highest educational level (N = 21,145**)		
Primary education or less	8231	38.9
Secondary or Higher	12,914	61.1
**Religion (N = 21,145)**		
Hindu	16,611	78.6
Others	4534	21.4
**Wealth index (N = 21,145)**		
Poor	14,188	67.1
Rich	6957	32.9
**Maternal age (N = 21,145)**		
15–24	11,063	52.3
25–34	9147	43.3
35–49	935	4.4
**Caste (N = 21,145)**		
Scheduled Caste	45	21.4
Schedule Tribe	2428	11.5
Other Backward Class	9265	43.8
Others	4932	23.3
**Sex of the child (N = 21,145)**		
Male	10,956	51.8
Female	10,189	48.2

Based on the intersectionality framework, individual social categories were combined to create an intersectional matrix (Table 3). Out of 21,145 children in the study, 12% belonged to the most advantaged group, i.e., children born to educated mothers from wealthy, Hindu families residing in urban areas. On the other hand, 6.6% of children belonged to the most disadvantaged group, i.e., children born to less educated mothers belonging to poor households from rural areas.

**Table 3:** Distribution of intersecting social categories

Intersecting social group (*N* = 21,145)	Frequency (*n*)	Percentage (%)
Urban-Secondary-Rich-Hindu (Most advantaged)	2562	12.1
Urban-Secondary-Rich-Others	887	4.2
Urban-Secondary-Poor-Hindu	621	2.9
Urban-Secondary-Poor-Others	221	1.0
Urban-Primary-Rich-Hindu	240	1.1
Urban-Primary-Rich-Others	182	0.9
Urban-Primary-Poor-Hindu	604	2.9
Urban-Primary-Poor-Others	233	1.1
Rural-Secondary-Rich-Hindu	2147	10.2
Rural-Secondary-Rich-Others	548	2.6
Rural-Secondary-Poor-Hindu	4996	23.6
Rural-Secondary-Poor-Others	932	4.4
Rural-Primary-Rich-Hindu	247	1.2
Rural-Primary-Rich-Others	145	0.7
Rural-primary-Poor-Hindu	5194	24.6
Rural-Primary-Poor-Others (Most disadvantaged)	1387	6.6

### Prevalence of exclusive breastfeeding of children below six months

**Fig. 1:**
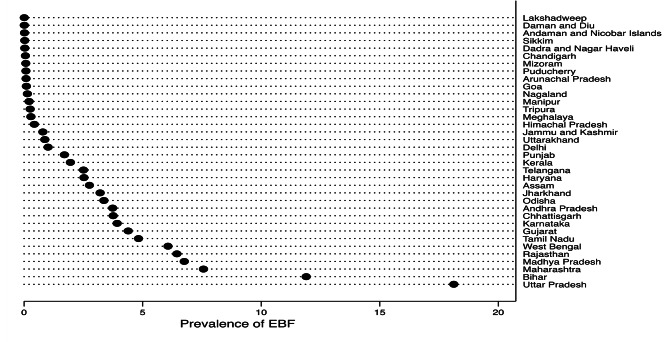
State-wise distribution of EBF prevalence

Overall, about 55% of the children (0–5 months) were exclusively breastfed in India. The highest prevalence was observed in the Northeastern regions of India (61.7%) and the lowest in the Central region (50.5%) (Table 4). The highest prevalence was reported in Uttar Pradesh, whilst the lowest was observed in Lakshadweep (Fig. 1). The state-wise prevalence of EBF varied substantially between the most advantaged **(**Urban Secondary Rich Hindu) and disadvantaged groups **(**Rural Primary Poor Others) (Table 5).

**Table 4:** Prevalence of exclusive breastfeeding of children below six months

Region	Exclusive breastfeeding	
	Yes (*n*%)	No (*n*%)
Northern India (N = 2738)	1529 (55.8)	1209 (44.2)
Southern India(N = 3618)	2083 (57.6)	1535 (42.4)
Eastern India(N = 5188)	2910 (56.1)	2278 (43.9)
Western India(N = 2554)	1440 (56.4)	1114 (43.6)
Central India (N = 6234)	3149 (50.5)	3985 (49.5)
North eastern India (N = 812)	501 (61.7)	311 (38.3)
**India (N = 21,145)**	**11,612 (54.9)**	**9533 (45.1)**

**Table 5:** Gap in EBF between most advantageous and least advantageous groups by states in India

State	EBF % in most advantaged group (Urban Secondary Rich Hindu)	EBF % in most disadvantaged group (Rural Primary Poor Others)*
Andhra Pradesh	55.1	88.0
Assam	68.0	60.2
Bihar	45.6	55.2
Chhattisgarh	76.8	80.0
Delhi	54.0	-
Gujarat	49.2	45.0
Haryana	48.9	57.1
Himachal Pradesh	60.0	-
Jammu & Kashmir	50.0	61.1
Jharkhand	56.3	64.2
Karnataka	43.0	40.0
Kerala	51.4	-
Madhya Pradesh	55.6	61.9
Maharashtra	57.8	50.0
Odisha	72.2	71.4
Punjab	55.2	50.0
Rajasthan	58.1	47.8
Tamil Nadu	48.8	-
Telangana	65.1	-
Uttar Pradesh	38.7	41.9
Uttarakhand	55.6	55.6
West Bengal	74.7	43.0
North-eastern states other than Assam	58.3	40.4
Union territories (UT) & Goa	54.5	-

*No child belonged to the most disadvantaged social category in some states/UT

### The pattern of intersecting inequalities in exclusive breastfeeding of children below six months

Table 6 presents the effect of intersecting categories on the EBF of children below six months. The logistic regression models were found to pass the Pearson goodness of fit test. The reference category was the most disadvantaged group. In both the crude and adjusted analyses only six out of 16 intersecting groups differed significantly from the reference category with the majority being within the rural intersecting categories. Rural-Primary-Poor-Hindus were 1.13 (95% CI 1.001, 1.277) times more likely to practice EBF compared to Rural-Primary-Poor-Others. Rural-Primary-Rich-Others were 0.49 (95% CI 0.345, 0.708) times less likely to be breastfed than the reference group. Rural-Secondary-Poor-Others were 1.21 (95% CI 1.024, 1.437) times more likely to practice exclusive breastfeeding than the reference group. Rural-Secondary-Poor-Hindus was 1.18 (95% CI 1.043, 1.332) more likely to practice EBF than the Rural-Primary-Poor-Others. Rural-Secondary-Rich-Hindus were 1.2 times (95% CI 1.042, 1.371) more likely to practice exclusive breastfeeding than Rural-Primary-Poor-others. Urban-Primary-Rich-Others were 0.53 times (95% CI 0.385, 0.733) less likely to practice exclusive breastfeeding than the reference group.

**Table 6:** Intersecting social position and exclusive breastfeeding of children

Intersecting categories	Prevalence of EBF within categories	cOR (95% CI)	*P*-value	aOR^1^ (95% CI)	*P*-value
Urban-Secondary-Rich-Hindu (N = 2562)	1384 (54.0)	1.096 (0.962, 1.250)	0.17	1.109 (0.972, 1.265)	0.13
Urban-Secondary-Rich-Others (N = 887)	465 (52.4)	1.030 (0.870, 1.220)	0.73	1.046 (0.883, 1.239)	0.60
Urban-Secondary-Poor-Hindu (N = 621)	311 (50.1)	0.940 (0.778, 1.137)	0.53	0.891 (0.736, 1.079)	0.24
Urban-Secondary-Poor-Others (N = 221)	128 (57.9)	1.219 (0.912, 1.631)	0.18	1.184 (0.884, 1.555)	0.26
Urban-Primary-Rich-Hindu (N = 239)	114 (47.7)	0.849 (0.645, 1.117)	0.24	0.829 (0.629, 1.093)	0.18
Urban-Primary-Rich-Others (N = 181)	65 (35.9)	0.526 (0.382, 0.725)	**< 0.01**	0.531 (0.385, 0.733)	**< 0.01**
Urban-Primary-Poor-Hindu (N = 604)	312 (51.7)	1.000 (0.826, 1.211)	1.00	0.946 (0.70, 1.147)	0.57
Urban-Primary-Poor-Others (N = 233)	111 (47.6)	0.856 (0.648, 1.130)	0.27	0.883 (0.668, 1.166)	0.38
Rural-Secondary-Rich-Hindu (N = 2147)	1213 (56.5)	1.210 (1.056, 1.385)	**< 0.01**	1.195 (1.042, 1.371)	**0.01**
Rural-Secondary-Rich-Others (N = 548)	279 (51.0)	0.947 (0.801, 1.190)	0.59	0.947 (0.776, 1.157)	0.60
Rural-Secondary-Poor-Hindu (N = 4996)	2865 (57.3)	1.257 (1.115, 1.416)	**< 0.01**	1.179 (1.043, 1.332)	**0.01**
Rural-Secondary-Poor-Others (N = 932)	537 (57.6)	1.266 (1.071, 1.497)	**< 0.01**	1.213 (1.024, 1.437)	**0.03**
Rural-Primary-Rich-Hindu (N = 247)	138 (55.9)	1.178 (0.897, 1.547)	0.24	1.137 (0.864, 1.495)	0.36
Rural-Primary-Rich-Others (N = 145)	50 (34.5)	0.495 (0.346, 0.708)	**< 0.01**	0.494 (0.345, 0.708)	**< 0.01**
Rural-Primary-Poor-Hindu (N = 5194)	2923 (56.3)	1.201 (1.066, 1.352)	**< 0.01**	1.131 (1.001, 1.277)	**0.05**
Rural-Primary-Poor-Others (N = 1387) (Reference)	717 (51.7)	1		1	

^1^Model was adjusted for maternal age, sex of child, and cast, cOR- Crude odds ratio, aOR- Adjusted odds ratio

**Fig. 2:**
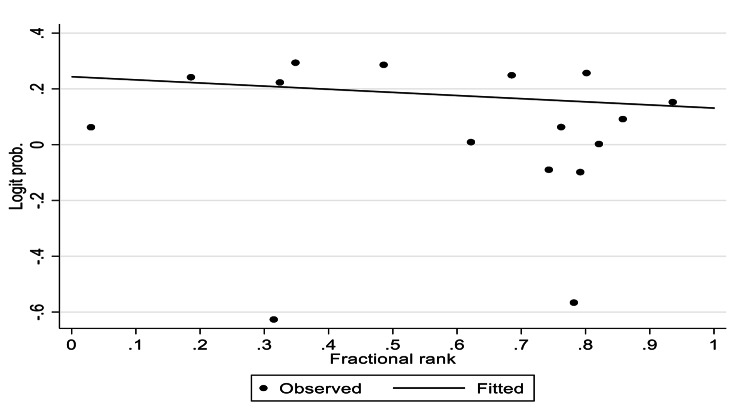
Slope inequality index graph

The absolute inequality, which is the difference in exclusive breastfeeding prevalence between the most advantaged and the most disadvantaged group, was only 2.3%. The relative disparity, i.e., ratio of EBF prevalence in both groups, was 1.04. The estimated relative inequality index was 0.95 (95% CI 0.89, 1.01, *p* - value < 0.01) whereas the slope inequality index (Fig. 2) was − 0.028 (95% CI -0.063, 0.001, *p* – value = 0.122). This indicated the clustering of the exclusive breastfeeding prevalence amongst the disadvantaged.

## Discussion

The present study intended to understand whether intersectional inequality exists in exclusive breastfeeding or not. Findings for this study indicate that intersecting inequalities exist in EBF based on religion, education of the mother, social class (wealth status), and place of residence. Factors like being from rural areas, higher education of mothers, and the Hindu religion seem to have positively affected exclusive breastfeeding prevalence whilst higher wealth index and urban living were likely to diminish the EBF of children below six months.

Although the most deprived group was Rural-Primary-Poor-Others, and the most advantaged group was Urban-Secondary-Rich-Hindus, the lowest and highest prevalence of EBF was reported amongst Rural-Primary-Rich-Others and Urban-Secondary-Poor-Others groups respectively. Also, the highest statistically significant odds of EBF were reported amongst rural categories (Rural-Secondary-Rich-Hindu, Rural-Secondary-Poor-Hindu, Rural-Secondary-Poor-Others, Rural-primary-Poor-Hindu). This finding seems to highlight that children living in rural households are most likely to be exclusively breastfed for the first six months than those living in urban areas. This observation was in line with the findings from other epidemiological studies which found EBF prevalence to be the highest in rural areas [18, 25–27]. The observed increased odds of exclusive breastfeeding amongst poor households from rural areas irrespective of other factors can be attributed to the high reliance on EBF as the primary source of nutrition for children within such households due to the inability of mothers to purchase infant formulas. In addition, since most of the nutrition and health education components of different national programs such as Integrated Child Development Services and Reproductive, Maternal, Neonatal, Child, and Adolescent Health Programmes (RMNCH + A) are targeted to poor rural households, it is more likely that children in such households will be exclusively breastfed [16].

The increased odds (albeit not significant) observed amongst some of the urban groups, seem to point out that given some contextual factors, EBF prevalence may be higher among some unique urban populations. For instance, the odds of exclusive breastfeeding although not significant was higher amongst the urban-secondary-poor-others group in comparison to the reference group (OR 1.184; 95% CI 0.884, 1.555, *p* - value = 0.26). Comparing this group to the reference group (Rural-Primary-Poor-Others), besides location, they also differ in maternal educational level. It can be inferred that given a better educational status, mothers in urban areas are more likely to engage in exclusive breastfeeding.

In contrast to this, the study results indicated that children born in either rural or urban areas and were in the primary-rich-others category were at significantly low odds of being exclusively breastfed. This decrease may be attributed to the negating effect of low maternal educational level, high household wealth status, and belonging to religions besides Hinduism irrespective of place of residence. Various studies carried out within the Indian population have reported that the above-listed demographic factors pose a negating effect on EBF amongst children [27–29]. For instance, mothers from affluent households have been reported to be less likely to practice EBF due to the high usage of various breast milk substitutes [30–33]. In addition, the poor in urban areas who often live in urban slum settlements have reduced health access to various breastfeeding initiatives and health education since they are often ignored when it comes to health policy action in urban areas [27, 28].

Compared to people who were in the least advantaged group (Rural-Primary-Poor-Others), children within the Rural-Primary-Poor-Hindu group were at increased odds of being exclusively breastfed. These groups were identical except for religious differences. The Hindu religion probably acted as a protective factor for exclusive breastfeeding as reported in previous studies from India and Nepal [16, 17, 34]. According to Hindu beliefs, breastmilk is the ideal food for newborns and breastfeeding is almost universal among Hindus.

The overall inequality seems to be less when considering exclusive breastfeeding prevalence amongst the extreme groups (the most advantaged and disadvantaged), however, this misses out on EBF prevalence amongst the middle groups. The slope and relative indices highlight the higher EBF prevalence amongst disadvantaged groups, albeit there exist wide disparities across the intersecting groups. This has huge implications from the policy perspective as most of the programs decide the beneficiaries without considering the probability of combined vulnerability or privilege. It is also essential to investigate the infant survival pattern in these 16 groups to determine the effect of inequality in exclusive breastfeeding practice.

### Strengths and limitations

This is the first study in India that tried to understand the inequality in EBF from an intersectionality perspective. The study also relied on large representative sample data with a high response rate, which makes the finding generalizable within the Indian context. The quantitative analysis of intersectional inequality is still an evolving area, and the present study offers a new methodological approach to the same. The limitations of secondary research, in general, are applicable to the present study.

## Conclusions

This research tried to understand and explain the critical question related to inequalities that existed in exclusive breastfeeding among infants less than six months in India. The results provide preliminary evidence on how the intersection of religion, place of residence, education, and social class inequalities influence EBF prevalence amongst children in India. It is vital to acknowledge this inequality and design targeted interventions that overcome the disadvantages and exploit the advantages to promote the EBF of children below six months. While within the general population, the WHO target of 50% exclusive breastfeeding prevalence amongst children under six months has been met, the prevalence of EBF remains low among some of the intersecting subgroups. There is therefore the need to improve breastfeeding practices in order to ensure this target is met by 2025.

In this regard, we recommend that existing programs and policies on exclusive breastfeeding should be evaluated for their inclusiveness since a majority often adopt a one-blanket approach without addressing the specific needs of people in different social positions. In order to overcome this hurdle, policies and programs seeking to improve EBF practice must adopt a multifaceted approach in the implementation, including the reallocation of financial resources based on the needs of the specific social groups. Further studies adopting qualitative methods are required to explore the facilitators and barriers of mothers within the intersecting groups to practice exclusive breastfeeding. This will provide an in-depth understanding of the process of intersectionality in exclusive breastfeeding practice and help design tailor-made interventions addressing the needs of such groups.

## Data Availability

The National Family Health Survey-4 (Demographic and Health Survey) datasets were available in the public domain (https://dhsprogram.com/data/available-datasets.cfm).

## Abbreviations

CEB: Census Enumeration Blocks
CI: Confidence Interval
EBF: Exclusive Breastfeeding
NFHS: National Family Health Survey
OR: Odds Ratio
PSU: Primary Sampling Units
